# Enactive account of pretend play and its application to therapy

**DOI:** 10.3389/fpsyg.2015.00175

**Published:** 2015-03-02

**Authors:** Zuzanna Rucinska, Ellen Reijmers

**Affiliations:** ^1^Department of Philosophy, University of Hertfordshire, Hatfield, UK; ^2^Interactie-Academie VZW, Antwerp, Belgium

**Keywords:** enactivism, pretend, play, systemic therapy, dialog

## Abstract

This paper informs therapeutic practices that use play, by providing a non-standard philosophical account of pretense: the enactive account of pretend play (EAPP). The EAPP holds that pretend play activity need not invoke mental representational mechanisms; instead, it focuses on interaction and the role of affordances in shaping pretend play activity. One advantage of this re-characterization of pretense is that it may help us better understand the role of shared meanings and interacting in systemic therapies, which use playing to enhance dialog in therapy rather than to uncover hidden meanings. We conclude with bringing together findings from therapeutic practice and philosophical considerations.

## INTRODUCTION

This paper explores one relationship between philosophical understanding of pretend play and therapies that include symbolic play with objects in their repertoire.

In traditional therapies (and particularly psychodynamic therapies), play has been used to “uncover” problems of clients to allow therapists to “analyze” them. In those therapies, play in general (pretend play at most, but also playing with objects) is often seen as symbolizing. Similarly, in philosophical works, pretend play, traditionally seen as symbolic play, is often characterized as a representational capacity whereby an object or behavior “stands in for” or represents another (see Mitchell, 2002). Mental representational structures dominate both the characterization and explanations of pretense activities. Such description of pretend play goes hand in hand with how playing is seen in therapy, which is as representing or denoting something true about the person who is playing.

Systemic therapy, however, is an approach that tends to focus on interaction and maintaining a dialog between a therapist and a client (Watzlawick et al., 1967; Watzlawick and Jackson, 2009). It asks for a different view of play, in which it is not a tool for uncovering and interpreting meanings, but is seen as part of a “here and now” dialog that allows discovering new meanings with a client in order to facilitate his/her development of novel perspectives. Likewise, the novel enactive account of pretend play (EAPP) proposes such a view of play. Based on the functional–ecological approach to pretense (Szokolsky, 2006), vast literature about the importance of interacting with objects in development of cognition and in establishing pretense relationships (e.g., Piaget, 1962; Vygotsky, 1978), and motivated by the emergence of novel embodied, intersubjective and (radically) enactive approaches to cognition (Gallagher, 2009; Hutto and Myin, 2013), the EAPP highlights the role of interaction in pretense. It further focuses on the key role that the notion of *affordances* may serve in shaping pretense activities when playing with objects (and other people), and suggests that even symbolic play need not invoke mental representations (Rucinska, 2014a,b). The advantage of the EAPP is that it looks better placed to provide an understanding of the role shared meanings and interacting serves in therapy that uses play. As such, it may fit better with the goals of systemic therapies, which focus on interaction.

In this article we aim to show that we can broaden the scope of what playing may be used for in therapy. We suggest a different function for engaging in play in therapy: one of creating a dialog, instead of being a mirror of reality. The EAPP gives further reasons why the “staying within play” approach (Rucinska and Reijmers, 2014) is beneficial in therapy, as it already pays special attention to engagements (active exploration of objects in relevant intersubjective contexts), finding mentality in the interactions and not in encapsulated mental representations.^1^ Understanding the possibility of a different account of pretend play as proposed by the EAPP makes an interesting case for therapists to reflect on their therapeutic practice.

## PLAY IN THERAPY

In traditional therapies (particularly psychodynamic therapies), play has been used to “uncover” problems of clients to allow therapists to “analyze” them. Drawing, playing with building blocks or puppets as well as pretend playing and role-playing is often used in various therapies, but as Russ and Fehr (2013) point out, “play therapy continues to be most associated with psychodynamic approaches.” In these approaches, play expressions are seen as manifestations of hidden or repressed longings, fears, or conflictive attachments, and are to be interpreted by the psychoanalyst. Verbalization and active labeling of the feelings are said to help the child understand and deal with the causes of their feelings and behavior (Freud, 1966; Dolto, 1985; Axline, 1989).

The psychodynamic approaches to play therapy are the dominant, but not the only available approaches. In systemic therapies, for example, play is used as a vehicle for communication and enhancing the dialog between the therapist and the client(s). The focus on interaction and communication in systemic therapy (see Watzlawick et al., 1967; Bateson, 1972; Watzlawick and Jackson, 2009) asks for a different view of play, seen as communication in context, and not as an expression of individual behaviors, thoughts or feelings that are projected onto the play or play materials, as in more traditional play therapy theories. The focus is not on what the play means or what the play expressions stand for, but on how the therapist can engage with the client in play in such a manner that it will enable the client’s change or shift of perspectives. As such, playing gives the therapist a new role.

We will highlight two challenges for systemic therapists that have to do with seeing therapy as such dialog. The first is to hold on to a “not-knowing stance” (Anderson, 2005, 2012). The second is *not* to attribute fixed meanings to play. Both challenges have to do with the therapists’ pitfalls to want to analyze play from outside of play, and to be in an expert position detached from the interaction (Cecchin, 1987; Cecchin et al., 1992; Bertrando, 2007).

Maintaining a “not-knowing stance” may be challenging to the therapist because trying not to interpret play, especially violent play, is not easy. Extreme behavior of children during therapeutic sessions, for example, can create situations where a systemic therapist cannot see the play as an ongoing interaction and is tempted to seek foothold in a “knowing stance.” When a therapist is overwhelmed, he/she is likely to see a negative situation as a mirror of reality, not as a creation of a reality in an on-going dialog. On the basis of experience and intuition, but probably also under pressure of dominant play theories that stress the idea of play as individual expression of feelings and longings, a therapist may determine a person’s problems *ex ante*, without exploring them further. For instance, impressed by the destructive way a boy behaved at a therapeutic session, a therapist at the *Interactie Academie* made a direct and determinate link between the aggressive moments in the game and the absence of a father figure. The boy’s aggression was no longer seen as a meaningful part of the game, but was perceived as an ever-present personal trait. At that moment, the therapist lost her creativity and the game with its playfulness stopped. However, when the therapist decided to take a different approach and introduce role-playing (where the players choose their characters and negotiated their roles), there was a mutual engagement in the therapeutic session. It seems plausible to suggest that this positive effect was, in part, a result of not stigmatizing the boy’s behavior and attributing blame. This example shows that the knowing position of the therapist, linked with her interpretation of the boy’s aggression as a hidden longing, can block creativity in play, whereas her focus on play without interpreting it created a different dynamic.

A related challenge for therapists is *not* to attribute fixed meanings to play, but to understand that there is a variety of meanings that play can carry. Consider another case from our practice of a young boy playing with a dollhouse during one therapeutic session. A 9 years old boy tidied the house, correctly arranged its furniture, swept the floors and played the piano in the play. He did this without saying a word. Then, choosing carefully, he placed every object in one room of the dollhouse. Finally he locked that room, leaving only empty rooms. The play appeared to be finished. In this example, the therapist is again under the risk of searching for the meaning behind the boy’s play, using dominant therapeutic theories and culturally embedded stories to analyze it. The dollhouse can be taken to stand in for the boy’s home, or the play to stand in for his feelings toward his family, but one way or another, it is taken to represent an actual state of affairs.

We suggest a different approach to understanding play in therapy. In this case, the dollhouse need not stand in for the boy’s specific feelings or family relationships; we have no way of knowing whether the play refers to the boy’s home unless the boy explicitly says that the dollhouse is like his home. We suggest that therapists should not pay attention to *what could be* the hidden meanings behind play, but to *how* a client is playing at the time, and how the therapist can in turn play with a client to further influence and negotiate the play. We base this suggestion on the idea that the meaning of the play need not be seen as *hidden behind* the action, but as *being in* and *emerging out of* the action. Play—even pretend play—need not be seen as representing meanings, fixed by mental representations. To support this idea, we turn to the EAPP.

## THE ENACTIVE ACCOUNT OF PRETEND PLAY

In standard philosophical approaches, pretend play, traditionally seen as symbolic play, is often characterized as a representational capacity whereby an object or behavior “stands in for” or represents another. That is because pretense itself is taken to be a type of a mental state that enables one to act as if one thing was another. The recurring aspect that underlies present pretense theories [whether metarepresentationalist (Leslie, 1987), behaviorist (Perner, 1991; Lillard, 1994; Harris, 2000; Nichols and Stich, 2003), or intentionalist (Rakoczy et al., 2004, 2005) is the positing of mental representations. There are many ways to characterize mental representations, ranging from a stronger cognitivist reading in which mental representations involve internal symbol-processing mechanisms with semantic information-bearing structures that store mental contents (Leslie, 1987), to weaker, action-oriented representations or some form of motor representations (Wheeler, 2005). However, Leslie’s (1987, p. 414) definition seems to best capture the mentioned theoretical models of pretense: “The basic evolutionary and ecological point of internal representation must be to represent aspects of the world in an accurate, faithful, and literal way, in so far as this is possible for a given organism.” To explain the capacity to pretend, the theorists then postulate various kinds of internal cognitive mechanisms, which manipulate the veridical mental representations to create new *pretense* representations (albeit through different means) that direct pretend play (Leslie, 1987; Harris, 2000; see Nichols and Stich, 2000; for most elaborate mechanism).^2^

Such description of pretend play goes hand in hand with how playing is seen in therapy, which is as representing or denoting something true about the person who is playing. Presently we propose an alternative account of pretense, where higher cognitive capacities such as offline symbol swapping need not be invoked. The EAPP proposes that basic cases of pretend play like treating one object as another only requires active exploration of objects in a playful context, as supported by the theory of (social) affordances (Gibson, 1979; Noë, 2004; Chemero, 2009) and agents’ sensorimotor skills (O’Regan and Noë, 2001).

Affordances are to be understood as possibilities for action. To quote Noë (2004, p. 105), “Things in the environment, and properties of the environment, offer or afford the animal opportunities to do things (find shelter, climb up, hide under, etc.). (…) When you see a tree, you not only directly perceive a tree, but you directly perceive something up which you can climb.” As such, the immediate environment can solicit certain actions and resist others. Applied to understanding pretense, we can think of objects as affording novel possibilities in and through the play. These possibilities depend on the actor’s sensorimotor skills and dispositions, as well as on the object’s properties, and the novelty and creative use of objects emerge through their interaction. Setting the interaction in a playful context also provides further flexibility to the actions, affecting the use that the objects solicit.

There is still a great debate about what affordances actually *are*, that is, whether they count as relational properties or dispositions, or whether they are inherently social (elicited by interacting with other people) or canonical (elicited by a wider social context and narrative practices; see Costall, 2012).^3^ Nevertheless, they are useful alternative constructs, both in terms of philosophy and therapy, as affordances can take over some of the purposes mental representations were supposed to serve. It is likely that in acting upon a prop (like in the banana–phone game), the player does not act independently of what is seen, but is guided by the prop (banana) and perceives in action what the prop affords (calling by holding to ear). Thus, affordances are strongly related to our sensorimotor capacity to interact with objects; they are best understood as the possibilities of action that come about in the interaction, as suggested by the action–perception–action loop: acting in the world brings about new affordances that further shape how you perceive and act on the world.^4^

This view reflects earlier, ecological approaches to pretense, where the nature of cognition is seen as dynamic and fluid, flexible and adaptable, and “pretend play is an especially good example of the fluid and dynamically intertwined presence of perception, action and cognition” (Szokolsky, 2006, p. 82). While more work is required to secure the EAPP, we provide here a first attempt at showing its benefits and relevance to therapy.

## APPLYING THE NEW PLAY METHOD

The EAPP can help to understand how to counter the two problems of systemic therapy: refraining from attributing prescribed meanings to behaviors, and taking the not-knowing stance. The notion of affordance can be useful for understanding that objects may not “denote” meanings but instead can “create” meanings through affording flexible actions to the actor. Regarding the “not knowing stance,” following Costall, Szokolsky (2006, p. 68) explains: “Any object has an immense number of action possibilities, but these cannot be known in advance, in separation from the actor and the action.” As we cannot know in advance what the objects can solicit in play (as their meanings are relatively flexible when negotiated in interaction), we should not fix our interpretations on them.

Taking an affordance-based view could allow therapists to have a different way of making use of play in therapy sessions. Consider an example of the “staying within play” approach, which uses play as a dialog that enables creating new meanings (Rucinska and Reijmers, 2014). This approach relies on using objects to create a playful dialog and an embodied experience. For example, one client (John) was asked to pick an object that would stand for the problematic relationship he wanted to deal with (the object happened to be a flexible snakelike ornament) as well as to pick objects to stand for different feelings he had regarding this relationship (he picked a book, an eraser and a colorful flower for his feelings and a sharpener, a feather and a postcard for the feelings of his son). John was then asked to put every object somewhere in the room, giving it a place in relation to the snakelike figure. Afterward, the therapist started a dialog with John about the form, shape and colors of the snakelike ornament and the way other objects were placed around it. Further, the therapist asked John to reposition the objects, as well swap seats with the therapist, who inquired further about how the relationship between the objects made John feel, what arrangements made him feel comfortable, and what bodily and emotional changes did he experience when he moved the objects around.

John and the therapist stayed, so to speak, *in* the play situation and *in* the play language. While this did not mark the end of the therapy sessions, there was a clear positive gain stemming from this form of interactive communication and hands-on engagement with objects; as John mentioned afterward, “he enjoyed the session, felt less depressive, and had a more hopeful feeling about the relationship that troubled him.” We believe this method allowed John to “position” himself differently to the problem. It suggests a great impact of offloading the problem to the objects that one can literally manipulate (have a hands-on embodied experience with) that allows one to get new perspectives and shift own attitudes (for more details on John’s case, see Rucinska and Reijmers, 2014).

## CONCLUSION

In this article we have suggested a different function for playing in therapy: one of creating a dialog, instead of being a mirror of reality. It shows that a therapeutic conversation is more than words. Playing, as an embodied activity, adds and reinforces the narratives, allowing new meanings to be created through object use and interaction with the therapist. Thus, while the use of creative methods and play is not new to systemic therapy, we believe that in the case above play served a special role: not only did it enrich the repertoire of the therapist, but it also allowed an embodied dialog to emerge. In this dialog, objects did not serve as “stand-ins” to be further analyzed but, rather, meanings attributed to the objects were “offloaded” onto them to be further manipulated.

We also aimed to show that the EAPP, involving a concept of affordances, can help us further understand the effects of the “staying within play” approach. It can be useful for therapists to understand that the traditional way play is characterized (as representational) may be consequential and skew the focus of the therapy, as therapists tend to look for inherent meanings in play behaviors of clients and concentrate on what they symbolize. This takes away from focusing on the interaction itself, where new meanings and understanding between therapists and clients can emerge.

As mentioned earlier, dominant cultural and therapeutic narratives make it difficult to see interaction and use of objects in a play situation as a way of creating meaning; meaning is supposedly already established or assumed. Thus, if we were to *operationalize* what is going on in these therapies, we would introduce mental representations of the semantic kind. The practice of using play to “uncover meanings” of “suppressed feelings” would be best characterized as involving represented “meanings,” “inherent” in the subject, whereby theorizing about them would be the right kind of practice to get to the mental life of the client.

Acknowledging the possibility of non-representational pretense motivates careful consideration of how play in therapy is to be understood, and broadens the spectrum of possibilities for therapists to use play in their therapy sessions. While taking a representational stance is tempting, it is not a necessary move, as an alternative is present. What is safe to say is that there seems to be a good fit between the EAPP and systemic therapy, in the sense that both focus on the interaction, where they find mentality. As the EAPP clarifies, interaction is a basis for mentality and is already in some sense meaningful, and so no extra level of mentality may need to be “uncovered.” The EAPP also gives an alternative account to how, without focusing on interpretations and thinking counterfactual thoughts but through engagement with objects, the relevant changes in attitudes (shift of perspective) can come about.^5^

Ultimately, with this paper we have aimed at promoting more research of interdisciplinary kind to shed further light on the implications that theories (and theoretical jargon) may have onto practice, within and between various disciplines. We hope to invite further research to be done in psychotherapy and cognitive psychology from developmental as well as cultural perspectives, using the notion of affordances and the EAPP.

### Conflict of Interest Statement

The authors declare that the research was conducted in the absence of any commercial or financial relationships that could be construed as a potential conflict of interest.

## Footnotes

1 By “mentality,” we broadly refer to kinds of mental or cognitive aspects of life.

2 It can be argued that this notion of mental representation underlies even the commitments of other theorists of pretence aside Leslie. Even the so-called “behaviorists” and “intentionalists” to pretence, who say that pretending is “merely acting as if,” commit to the view that one is “acting as if” *a proposition is true*. For example, Harris (2000) claims that to successfully play banana–phone, a child must act according to a rule (or as he call it a “flag”) that “this banana is not a phone” and edit these rules to generate new flags through a propositional model, with “statements written on the various flags” (p. 66), while Rakoczy et al. (2005, p. 81) claim that “in pretending to pour the actor symbolizes ‘there is water coming out of this container,’ he acts as if it was true.” There is a clear indication that, explicitly intended or not, these theorists too commit to the notion of mental representation of the stronger, semantic kind.

3 For example, adults initiate and guide children’s play by showing how to play, which the child imitates, and encourage pretence play through various forms of verbal and nonverbal feedback. Immediate dialogical interactions afford others as potential co-operators. Intersubjective contexts can allow new ways of understanding to be established in the interaction [in what De Jaegher and Di Paolo (2007) call “participatory sense-making” activity]. Social context determines whether there is a breakdown in the play (such as when “flying movements” are used in “elephant” play) or whether it is accommodated (as “Dumbo the flying elephant” play). Such co-creation of meanings suggests that sensitivity to others’ understandings, stemming from engagements in joint activity, allow for new, shared understandings to develop.

4 That action and perception are tightly bound has been proposed and defended extensively in the literature (Noë, 2004), and can be seen in empirical findings. For example, Held and Hein’s (1963) famous “Kitten Carousel” experiment showed that there was a significant difference in how the active kitten, controlling its locomotion, responded to its environment (avoiding visual cliffs, bracing themselves from being placed on the visual cliff, or avoiding looming objects) as opposed to the passive kitten, which did not engage in such behaviors. This finding suggests that there is an action–perception–action loop, whereby the engagement in moving around afforded its avoidance of visual cliffs. Thus, quoting Chemero (2009, p. 145), it is “more appropriate to understand affordances as being inherent not in animals, but in animal-environment systems.”

5 That psychodynamic therapy mostly involves representational model should be further substantiated and tested against new developments within the wider field of psychodynamic therapy like the earlier-mentioned relational depth psychology approaches, which assign a significant importance to the “here-and-now” interpersonal realities unfolding within the therapeutic settings (see Stern, 2004). But while it is not excluded, for example, that there may be a way to accommodate psychodynamic approaches to uncovering meanings in therapy with the EAPP (under a different description of “meanings”), there seems to be a more natural fit between these approaches and the representational account of pretence.
